# The morphological and molecular characterization of *Baylisascaris devosi* Sprent, 1952 (Ascaridoidea, Nematoda), collected from Pine marten (*Martes martes*) in Iran

**DOI:** 10.1186/s13071-020-04513-4

**Published:** 2021-01-08

**Authors:** Meysam Sharifdini, Richard A. Heckmann, Fattaneh Mikaeili

**Affiliations:** 1 Department of Medical Parasitology and Mycology, School of Medicine, Guilan University of Medical Sciences, Rasht, Iran; 2 Department of Biology, Brigham Young University, 1114 MLBM, Provo, Utah 84602 USA; 3 Department of Parasitology and Mycology, School of Medicine, Shiraz University of Medical Sciences, Shiraz, Iran

**Keywords:** *Baylisascaris devosi*, *Martes martes*, Iran, Molecular characterization, SEM, EDAX

## Abstract

**Background:**

*Baylisascaris devosi* is an intestinal nematode found in several carnivores including fisher, wolverine, Beech marten, American marten and sable in different parts of the world, but this nematode has not been reported from Pine marten. Therefore, this study aimed to identify *Baylisascaris* isolated from a Pine marten in Iran using morphological and molecular approaches.

**Methods:**

Specimens of *B. devosi* were collected from one road-killed Pine marten in northern Iran. Morphological features were evaluated using scanning electron microscopy, energy dispersive x-ray analysis and ion sectioning. The molecular characterization was carried out using partial *Cox1*, LSU rDNA and ITS-rDNA genes.

**Results:**

The nematodes isolated from the Pine marten were confirmed to be *B. devosi* based on the morphological features and the sequence of ribosomal and mitochondrial loci. X-ray scans (EDAX) were completed on gallium cut structures (papillae, eggs, male spike and mouth denticles) of *B. devosi* using a dual-beam scanning electron microscope. The male spike and mouth denticles had a high level of hardening elements (Ca, P, S), helping to explain the chemical nature and morphology of the worm. Based on these genetic marker analyses, our sequence had the greatest similarity with Russian *B. devosi* isolated from sable.

**Conclusions:**

In this study, to our knowledge, the occurrence of *B. devosi* infection in Pine marten is reported for the first time. Molecular analysis showed that these three genes are suitable molecular markers for identification and inferring phylogenetic relationships of *Baylisascaris* species. Furthermore, the high divergence of *Cox1* between *Baylisascaris* species indicates that *Cox1* could be used for their phylogenetic and taxonomic studies. 
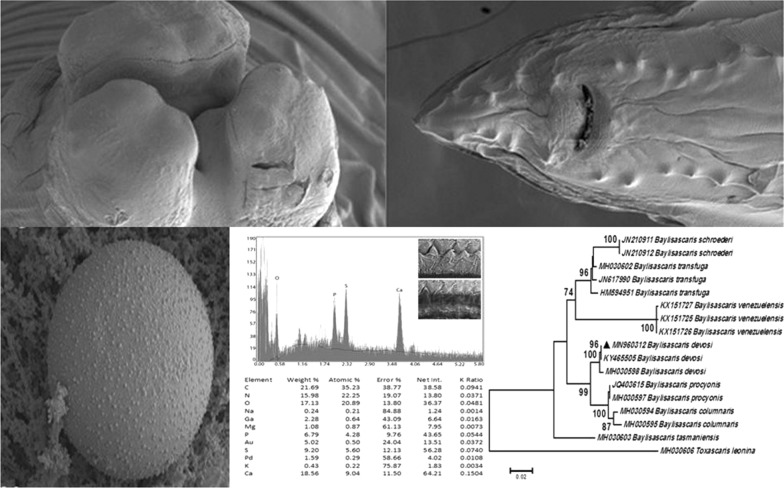

## Background

*Baylisascaris* is a roundworm belonging to the family Ascarididae that has several species, including *B. procyonis, B. melis, B. transfuga, B. columnaris, B. devosi, B. laevis, B. shroederi, B. venezuelensis* and *B. potosis.* The various species of *Baylisascaris* have specific definitive hosts and can be identified accordingly [1]. Unembryonated eggs are shed in the feces of the definitive host and become infective in the environment within several weeks. Definitive hosts can be infected by ingesting embryonated eggs from the environment or the consumption of encapsulated third-stage larvae in the muscles of paratenic hosts such as rodents and birds [2]. Human is an accidental host for this parasite, and baylisascariasis in human can be caused by *B. procyonis*, *B. columnaris*, *B. melis*, *B. devosi*, *B. transfuga* and *B. tasmaniensis* [3]. *Baylisascaris procyonis*, the Raccoon roundworm, is the most important species of *Baylisascaris* and the primary cause of baylisascariasis in human because the larval form is capable of causing severe neurological disease in human. Consequently, the other *Baylisascaris* species have been less studied compared to *B. procyonis* [4]. Since baylisascariasis is diagnosed by serological methods and these cannot identify the parasite species, molecular methods are useful for the identification of *Baylisascaris* species [3].

*Baylisascaris devosi* was first described as a new species of ascaridids from carnivorous mammals such as fisher (*Pekania pennant*) and Beech marten (*Martes foina*) such as *Ascaris devosi* [5]. Kontrimavichus (1963) considered ascaridids of mustelids to be *B. devosi* Sprent, 1952 [6]. Adult worms of *B. devosi* live in the intestinal tract of carnivorous mammals including fisher, wolverine (*Gulo gulo*), Beech marten, sables (*Martes zibellina*) and American marten (*Martes americana*) [5, 7].

The different species of *Baylisascaris* can be distinguished from each other based on morphological features, but since the morphological identification of *Baylisascaris* species is difficult, especially at the egg, larval and immature worm stages, molecular methods have been used for accurate identification. For the first time, *B. devosi* isolated from wolverine was identified using a molecular method along with the morphological technique, and the study suggested that both morphological and molecular methods are needed for accurate identification of *Baylisascaris* species [8]. In the other study, *B. devosi* nematodes collected from Kamchatka sables were identified based on morphological characteristics, and the molecular method was used to confirm their morphology-based identification [9]. In this study, for the first time to our knowledge, *B. devosi *has been isolated from Pine marten (*Martes martes*) in Iran; therefore, the current study was performed on identification of *Baylisascaris* based on morphological and molecular approaches. Furthermore, chemical analysis of structures of *B. devosi* was carried out using energy-dispersive x-ray analysis for the first time to our knowledge.

## Methods

### Collections

Seven adult nematodes, including four female worms and three male worms, were collected from one road-killed Pine marten in Ramsar district (36°47′N, 50°35′E), Mazanderan Province, northern Iran, in August 2019. The animal was an adult male weighing 1700 g, and morphometric measurements were: body length = 47 cm; tail length = 25 cm; shoulder height = 19 cm. A comprehensive autopsy was performed, and only the specimens of ascarid nematodes were collected from the intestine. The recovered worms were washed extensively in physiological saline. Two specimens were fixed in 70% (v/v) ethanol for transport to Brigham Young University in Utah, USA, for scanning electron microscopy (SEM) studies, metal analysis and gallium (Ga) sections. Also, two specimens were preserved in 70% ethanol until extraction of genomic DNA for molecular study.

### SEM (scanning electron microscopy)

Specimens that had been fixed and stored in 70% ethanol were processed for scanning electron microscopy (SEM) following standard methods [10]. These included critical point drying (CPD) in sample baskets and mounting on SEM sample mounts (stubs) using conductive double-sided carbon tape. Samples were coated with gold and palladium for 3 min using a Polaron #3500 sputter coater (Quorum (Q150 TES; www.quorumtech.com) establishing an approximate thickness of 20 nm. Samples were placed and observed in an FEI Helios Dual Beam Nanolab 600 (FEI, Hillsboro. Oregon) scanning electron microscope with digital images obtained in the Nanolab software system (FEI, Hillsboro, OR) and then transferred to a USB for future reference. Samples were received under low vacuum conditions using 10 KV, spot size 2, 0.7 Torr, using a GSE detector.

### Energy-dispersive x-ray analysis

Standard methods were used for preparation similar to the SEM procedure. Specimens were examined and positioned with the above SEM instrument, which was equipped with a Phoenix energy-dispersive x-ray analyzer (FEI, Hillsboro, OR). X-ray spot analysis and live scan analysis were performed at 16 Kv with a spot size of 5, and results were recorded on charts and stored with digital imaging software attached to a computer. The TEAM (Texture and Elemental Analytical Microscopy) software system (FEI, Hillsboro, OR) was used. Data were stored on a USB for future analysis. The data included weight percent and atom percent of the detected elements following correction factors.

### Ion sectioning

A dual-beam SEM with a gallium (Ga) ion source (GIS) is used for the LIMS (liquid ion metal source) part of the process. The structures (male spike, papillae, egg and mouth denticles) of the worm were centered on the SEM stage and cross sectioned using a probe current between 0.2 nA and 2.1 nA according to the rate at which the area was cut. The time of cutting is based on the nature and sensitivity of the tissue. Following the initial cut, the sample also underwent a milling process to obtain a smooth surface. The cut was then analyzed with an x-ray for chemical ions with an electron beam (Tungsten) to obtain an x-ray spectrum. Results were stored with the attached imaging software. The intensity of the Ga beam was variable according to the nature of the material being cut.

### DNA extraction and PCR amplification

For DNA extraction, adult worms of *B. devosi* were washed three times in distilled water to remove ethanol. Total genomic DNA was extracted using the Qiagen DNeasy tissue kit (Qiagen Inc., Valencia, CA, USA) according to the manufacturer’s instructions. Partial mitochondrial cytochrome c oxidase 1 (*Cox1*), large subunit ribosomal ribonucleic acid (LSU rDNA) and ITS-rDNA genes were subjected to PCR amplification. The forward primer LCO1490 (5′-GGTCAACAAATCATAAAGATATTGG-3′) and reverse primer HCO2198 (5′-TAAACTTCAGGGTGACCAAAAAATCA-3′) were used to amplify an about 700 bp fragment of the *Cox1* gene [11]; the forward primer LSU391 (5′- AGCGGAGGAAAAGAAACTAA- 3′) and reverse primer LSU501 (5′-TCGGAAGGAACCAGCTACTA- 3′) were used for the amplification of a 1100 bp fragment of the D2D3 expansion segment of LSU rDNA [12]. Also, a 1050-bp-long amplicon containing partial 18S rDNA, complete ITS1, 5.8S and partial ITS2 rDNA was amplified using the forward primer Vrain_F (5′ TTGATTACGTCCCTGCCCTTT-3′) and the reverse primer AB28 (5′ ATATGCTTAAGTTCAGCGGGT-3′) [13, 14]. All PCR reactions were carried out in a 30 μl reaction mix, containing 15 μl of PCR premix (2x Master Mix RED Ampliqon, Odense, Denmark), 20 pmol of each primer and 2 μl of template DNA. The temperature profile was one initial denaturation cycle at 95 ℃ for 4 min followed by 35 cycles of denaturation at 94 ℃ for 30 s, annealing at 55 ℃ for 30 s (for *Cox1*), 49 ℃ for 30 s (for LSU rDNA) and 52 ℃ for 35 s (for ITS-rDNA) and extended at 72 ℃ for 1 min, with a final extension step at 72 ℃ for 5 min. A sample containing water instead of template DNA was included in each run as a negative control.

The PCR products were separated by electrophoresis on a 1.5% agarose gel and visualized using a UV transluminator (Vilber Lourmat, Collégien, France). The amplification products were sequenced on an ABI 3730 automatic sequencer (Applied Biosystems, Foster City, CA, USA) in both directions, using the same PCR primers as used in the PCR.

The sequence results were edited and trimmed using Chromas v.2.01 and Geneious software (www.geneious.com). The basic local alignment search tool (BLAST) program (http://www.ncbi.nlm.nih.gov/blast/) was used to compare the consensus sequences with GenBank references sequences. The sequences obtained in this study were deposited in the GenBank database (accession numbers: MN960313 for the partial LSU rDNA; MN961617 for the partial *Cox1* gene; MN960312 for the ITS-rDNA gene).

### Phylogenetic analysis

Phylogenetic trees were constructed with sequences obtained in the present study along with reference sequences deposited in GenBank using the maximum likelihood (ML) method and Tamura-3 parameter model, and genetic distances were calculated with the maximum composite likelihood model in MEGA6 software (http://www.megasoftware.net/). The reliabilities of the phylogenetic trees were assessed using the bootstrap value with 1000 replications. The sequences used for the phylogenetic analysis are listed in Table 1.

**Table 1.** Geographic origin, host, accession numbers and references of *Cox1*, LSU rDNA and ITS-rDNA sequences of *Baylisascaris* species deposited in GenBank

Species	Host	GenBank acc. no. LSU rDNA	GenBank acc. no. ITS rDNA	GenBank acc. no. *Cox1*	Location	References
*B. devosi*	*Martes martes*	MN960313	MN960312	MN961617	Iran	Current study
*B. devosi*	*Pekania pennanti*	MG937776	MH030598	MH795151	Canada	[24]
*B. devosi*	*Martes zibellina*	KY465564	KY465505	KX646394	Russia	[9]
*B. procyonis*	*Procyon lotor*	MG937774 and MG937775	MH030597	–	USA	[24]
*B. procyonis*	*Procyon lotor*	AY821774	–	–	USA	[30]
*B. procyonis*	*Procyon lotor*	–	JQ403615	–	Norway	[31]
*B. procyonis*	*Procyon lotor*	–	MH030597	–	USA	[32]
*B. procyonis*	*Procyon lotor*	–	–	KJ698559, KJ698566 and KJ698567	China	Unpublished
*B. procyonis*	*Procyon lotor*	–	–	JF951366	China	[26]
*B. columnaris*	*Mephitis mephitis*	MG937772 and MG937773	MH030594 and MH030595		USA	[24]
*B. columnaris*	*Mephitis mephitis*	–	–	KY580736, KY580738 and KY580739	USA	[33]
*B. transfuga*	*Ursus americanus*	MH551546	–	–	Canada	[32]
*B. transfuga*	*Ursus americanus*	–	MH030602	–	USA	[24]
*B. transfuga*	*Ursus arctos*	MG937779	–	–	Canada	[24]
*B. transfuga*	*Ursus maritimus*	JN257008	–	–	China	[25]
*B. transfuga*	*Ursus arctos*	KC543471	–	–	The Netherlands	[34]
*B. transfuga*	Na^a^	–	JN617990	–	Na	Unpublished
*B. transfuga*	*Thalarctos maritimus*	–	HM594951		Italy	[35]
*B. transfuga*	*Ursus maritimus*	–	–	HQ671079	China	[26]
*B. schroederi*	*Ailuropoda melanoleuca*	JN257013	–	–	China	[25]
*B. schroederi*	*Ailuropoda melanoleuca*	–	–	KJ587808 and KJ587837	China	[36]
*B. schroederi*	*Ailuropoda melanoleuca*	–	–	HQ671081	China	[27]
*B. ailuri*	*Ailurus fulgens*	JN257012	–	–	China	[25]
*B. ailuri*	*Ailurus fulgens*	–	–	HQ671080	China	[27]
*B. tasmaniensis*	*Sarcophilus harrisii*	MG937781	–	–	Australia	[24]
*B. tasmaniensis*	*Sarcophilus harrisii*	–	MH030603	–	Australia	[32]
*B. venezuelensis*	*Tremarctos ornatus*	_	KX151725, KX151726 and KX151727	–	Venezuela	[37]

^a^Na, not available

## Results

### Morphological

**Fig. 1:**
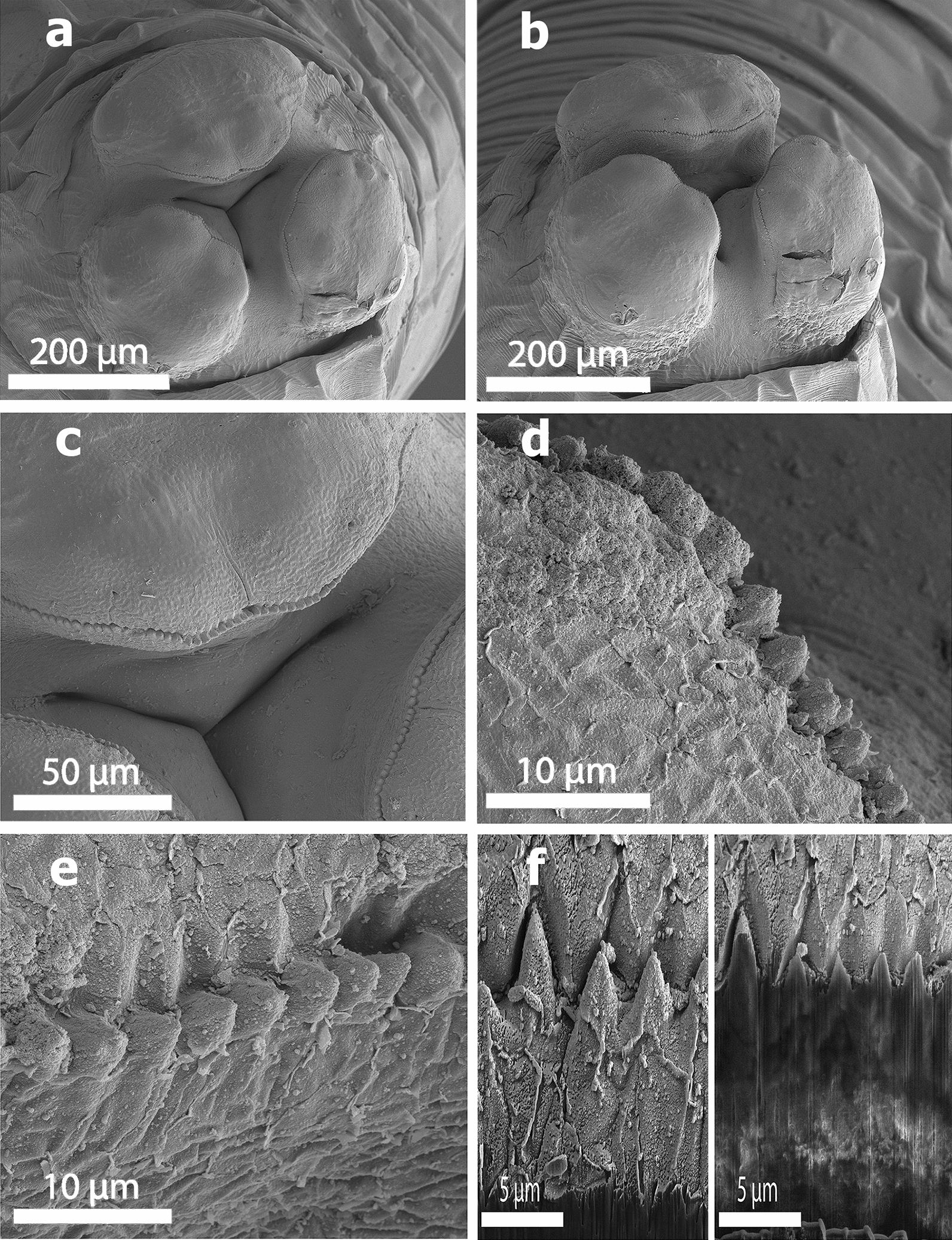
SEM of specimens of *Baylisascaris devosi* from *Martes martes* in Iran. **a** En face view of the mouth area with the three fleshy lips characteristic of Ascarid worms. **b** Lateral view of the mouth entrance for the worm. **c** One of the fleshy lips with teeth-like denticles on the surface. **d** High magnification of the denticles. **e** En face view of the denticles found on both surfaces of the lip. **f** Intact and cut denticles (see x-ray print-out of a cut denticle)

**Fig. 2:**
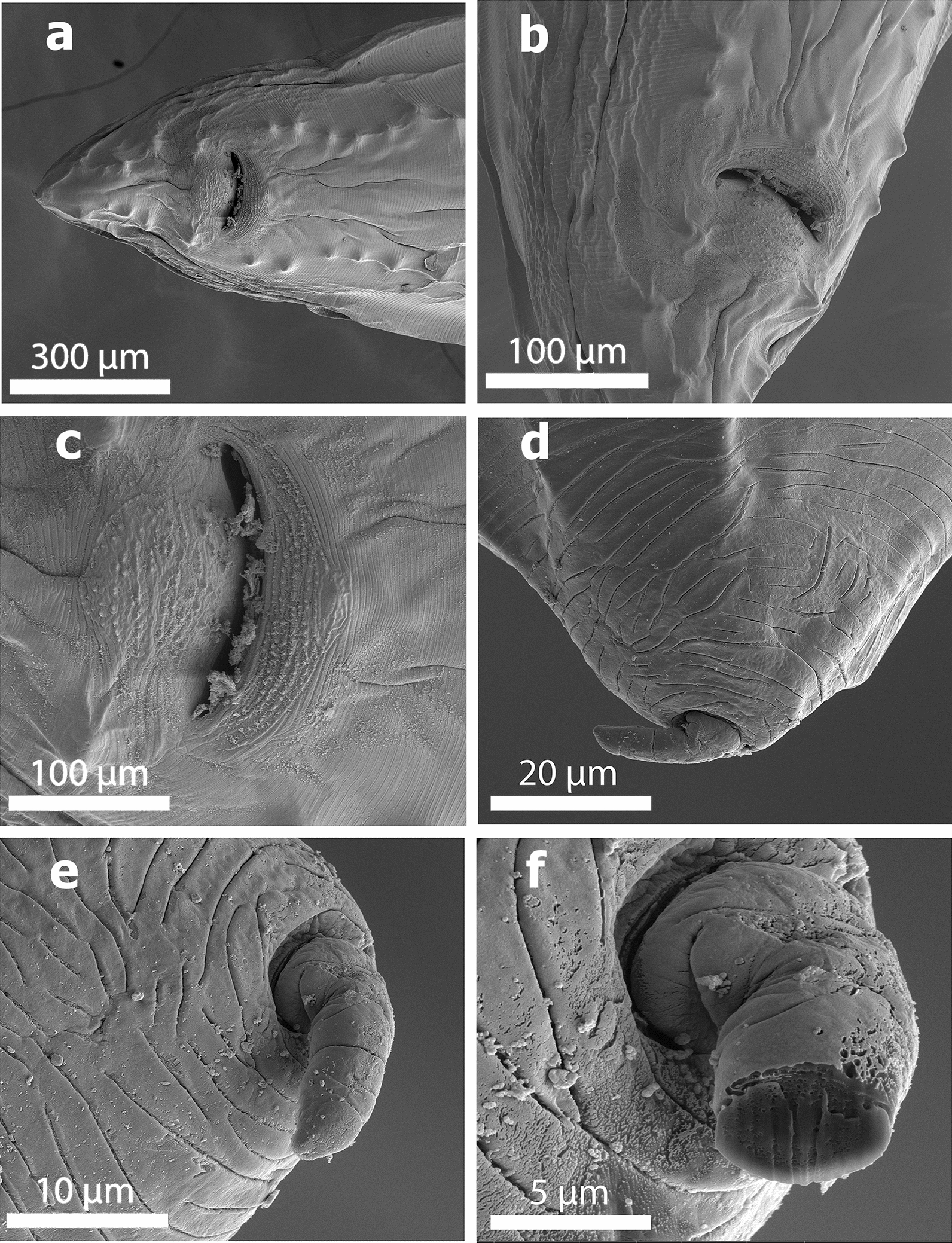
SEM of specimens of *Baylisascaris devosi* from *Martes martes* in Iran. **a** Cloacal region of the male ascarid worm with numerous papillae, the number of which is a taxonomic key. **b** Lateral view of the male ascarid worm with numerous papillae. **c** Cuticular structures around the male cloacal opening. **d**, **e** A small distinct spike found at the posterior end of the worm. **f** Intact and gallium cut of the male spike (see x-ray print-out for cut male spike)

**Fig. 3:**
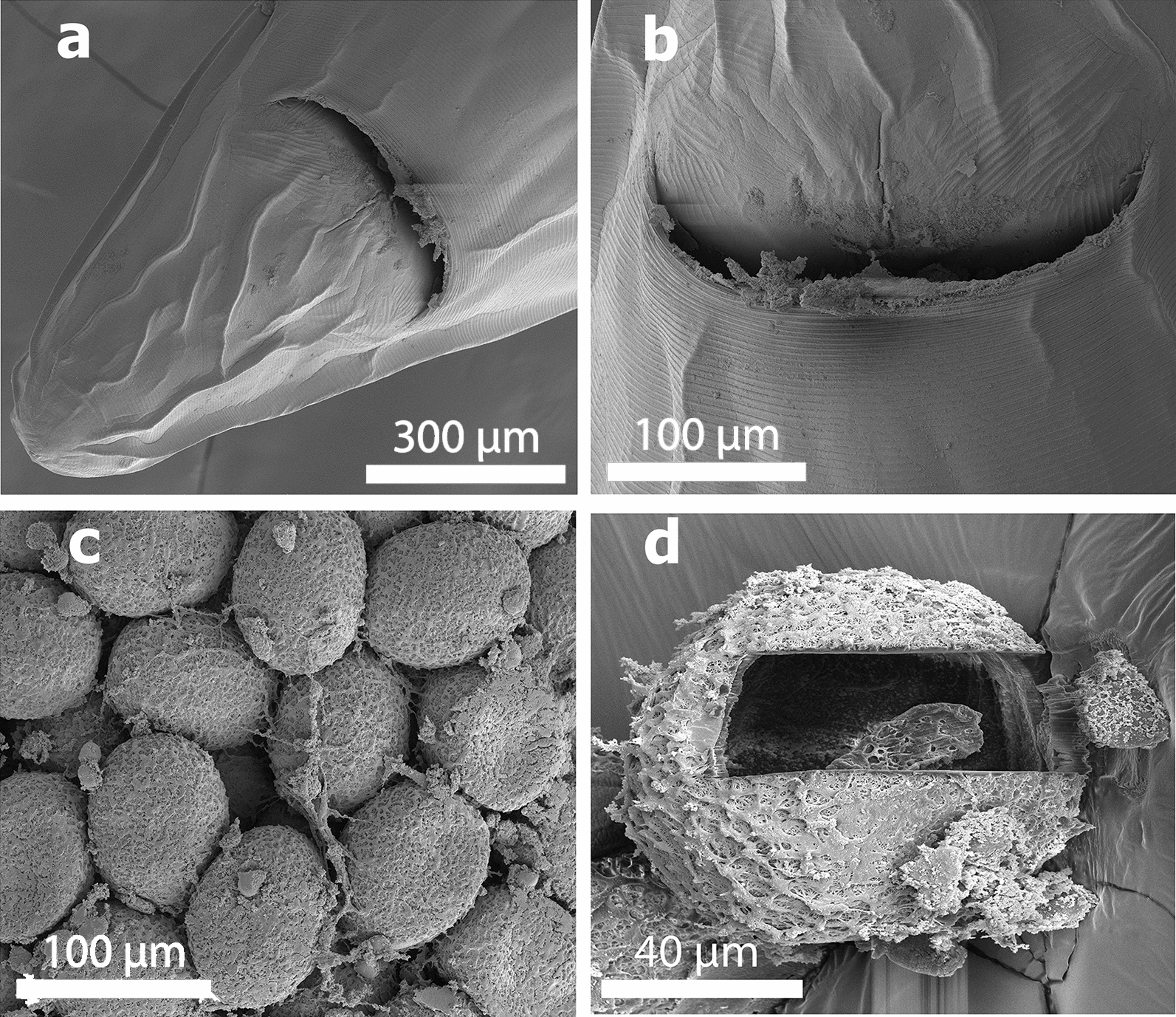
SEM of specimens of *Baylisascaris devosi* from *Martes martes* in Iran. **a** Posterior view (ventral side) of the female worm with the anal opening. **b** Smooth surrounding surface of female anal opening. **c** Eggs of *Baylisascaris devosi*. **d** Gallium cut egg of the worm (see x-ray print-out of the cut egg)

The body length of male and female worms was 6.5–12 cm (*n* = 3) and 8–16 cm (*n* = 4), respectively. Observation with the scanning electron microscope showed that the triangular mouth of *B. devosi* was surrounded by three lips; one of the lips was located in dorsal position and the other two in the ventral position (Fig. 1a, b). There was a pair of sensory papillae on the lateral margin of the lips, and the inner face of the free edge of each lip was armed with small denticles (Fig. 1c–f). The male posterior end showed the presence of pre- and post-cloacal papillae that scattered on the sub-ventral part. The total count of pre-cloacal papillae was 28 pairs. There were four pairs of post-cloacal genital papillae (Fig. 2a); the first and second pairs were double but the third and fourth pairs were single (Fig. 2a, b). There were cuticular structures around the cloacal opening in the male worm (Fig. 2c). Also, a small distinct spike was present on the posterior end of the tail (Fig. 2d–f). In the female worms, the vulvar opening was situated in the anterior half of the body length. The female anal opening had a smooth surrounding surface without any papillae (Fig. 3a, b). The fertilized eggs were ellipsoidal in shape and covered with minuscule pits (Fig. 3c, d).

**Fig. 4:**
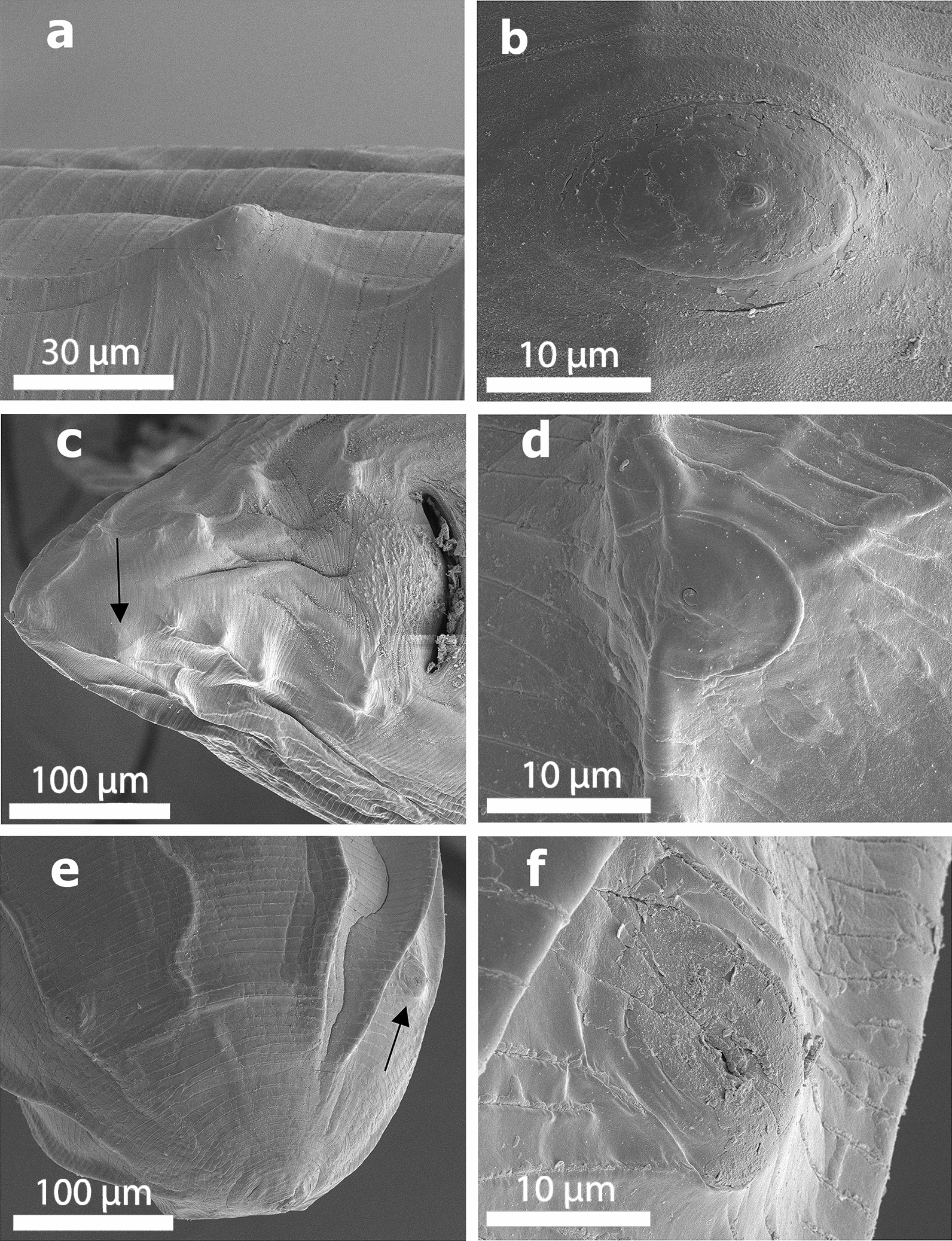
SEM of specimens of *Baylisascaris devosi* from *Martes martes* in Iran. **a** Lateral view of papillae on the cuticular surface of the male worm. **b** Enlarged male papillae. **c** Posterior part ventral side of male showing the phasmids (arrow) and other structures such as papillae. **d** High magnification of the phasmid of the male ascarid. **e** Posterior end of the female showing the phasmids (arrow). **f** Phasmid for the female ascarid

Figure 4a, b shows high magnification of the male papillae. The male phasmids were situated after the fourth pair of post-cloacal papillae (Fig. 4c, d). Phasmids of the female worm were located on the sub-ventral side of one-third of the posterior part of the tail (Fig. 4e, f).

### Energy-dispersive x-ray analysis (EDXA)

**Fig. 5:**
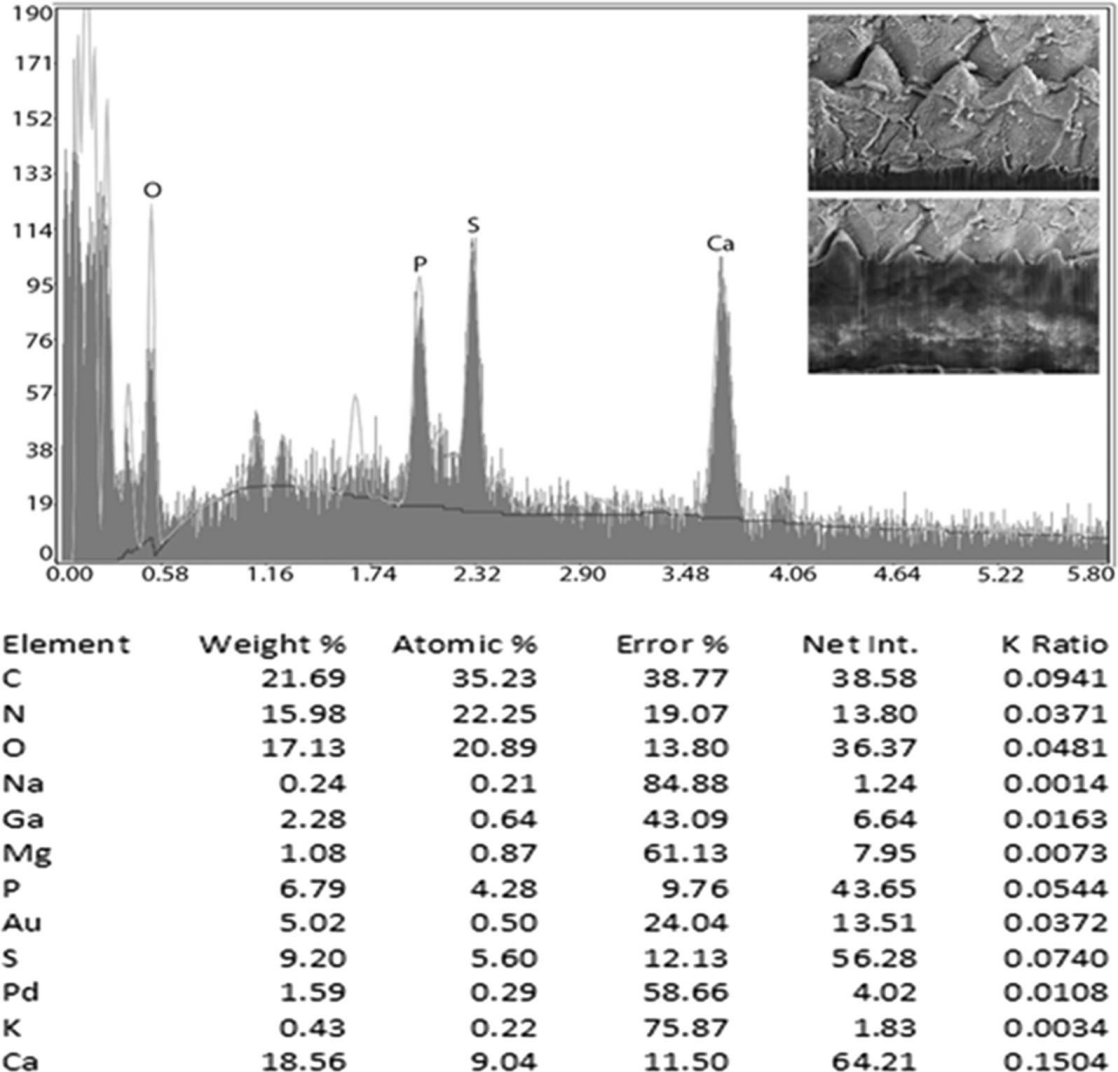
Energy-dispersive x-ray spectrum for the denticles found on surfaces of the lip of a *Baylisascaris devosi* specimen showing high levels of calcium, sulfur and phosphorus. Insert: SEM of a gallium cut denticle

**Fig. 6:**
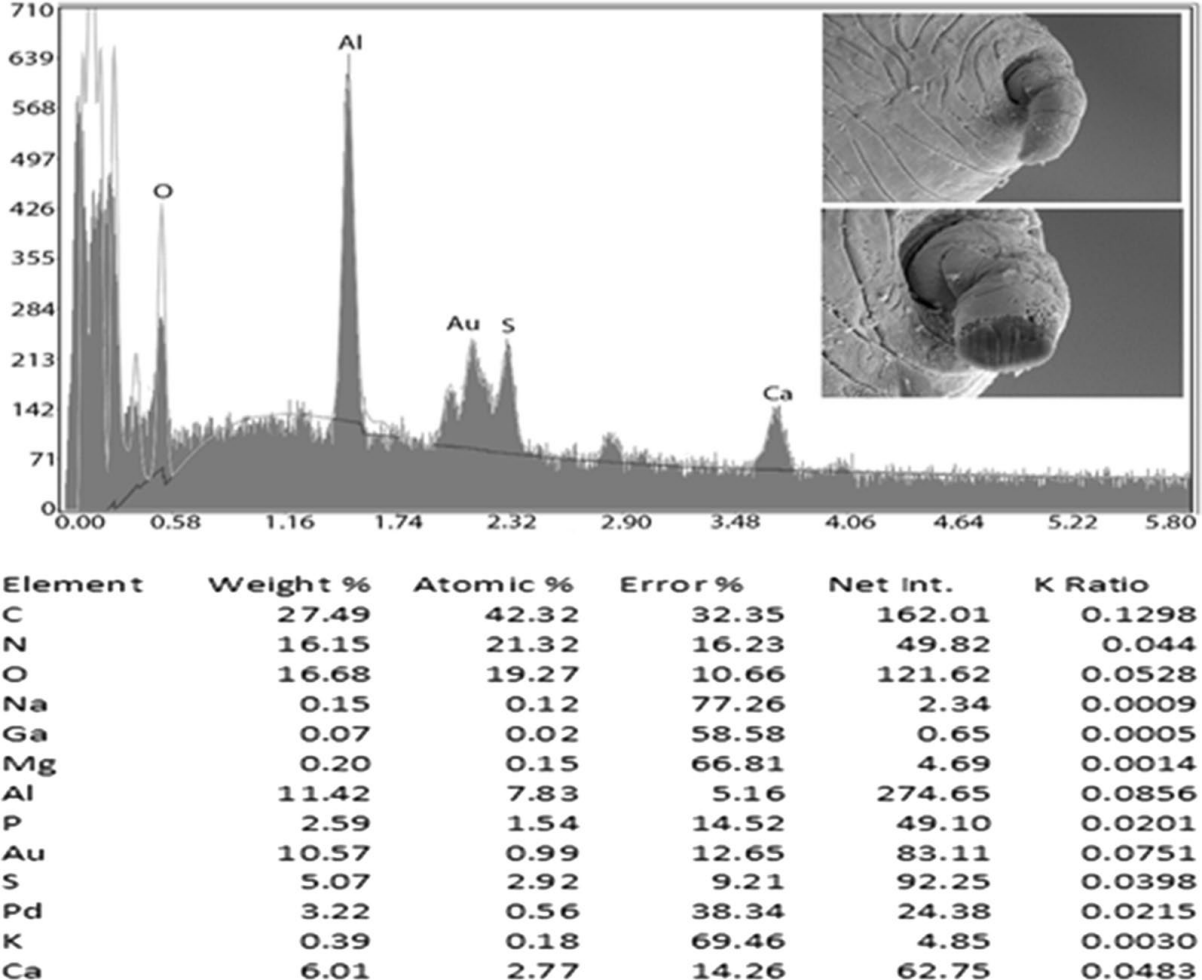
Energy-dispersive x-ray spectrum for the male spike of a *Baylisascaris devosi* specimen showing high levels of calcium and sulfur. Insert: SEM of a cross gallium cut male spike

**Fig. 7:**
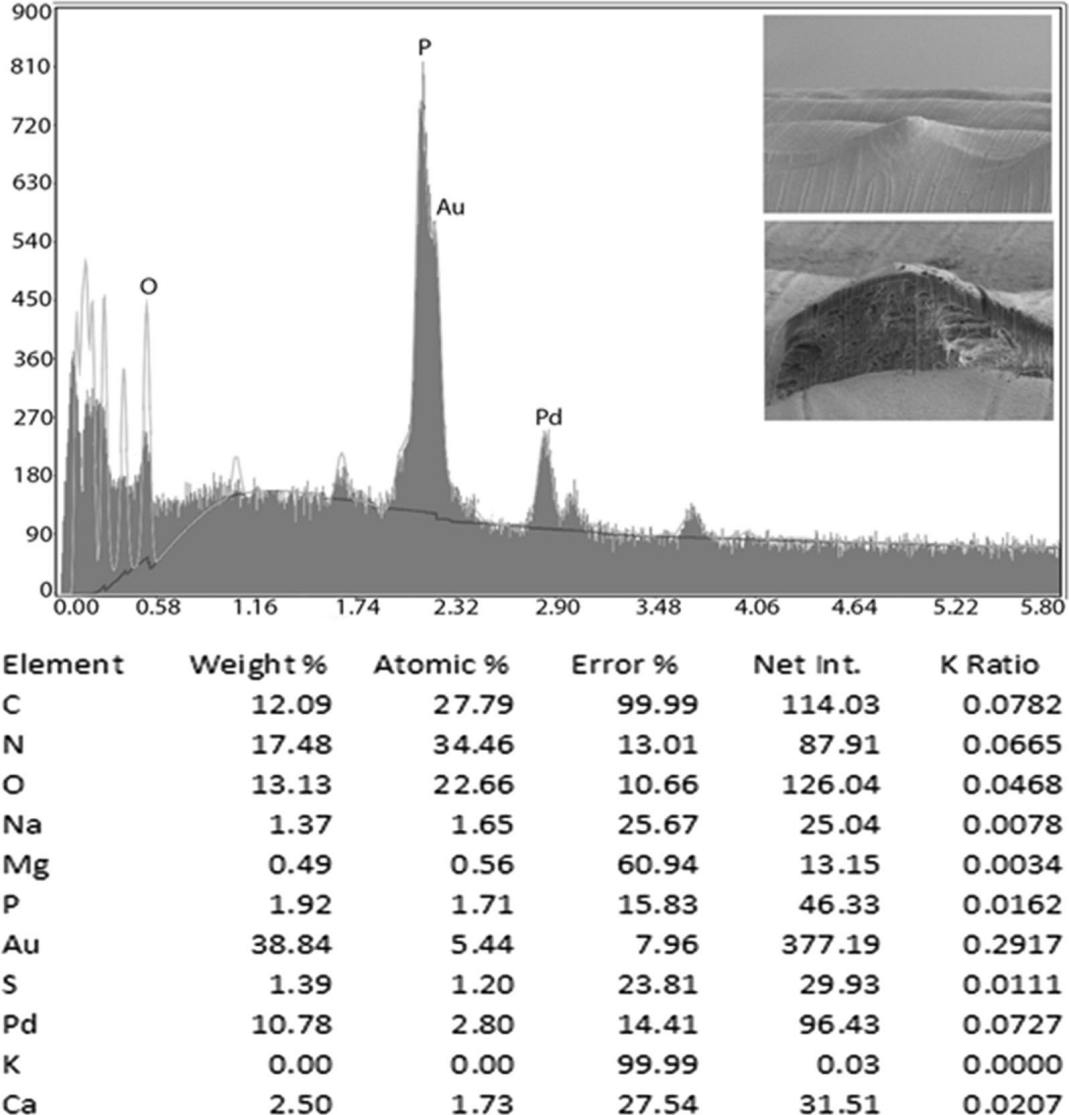
Energy-dispersive x-ray spectrum of the male papillae of a *Baylisascaris devosi* specimen. Insert: SEM of a gallium cut male papillae

**Fig. 8:**
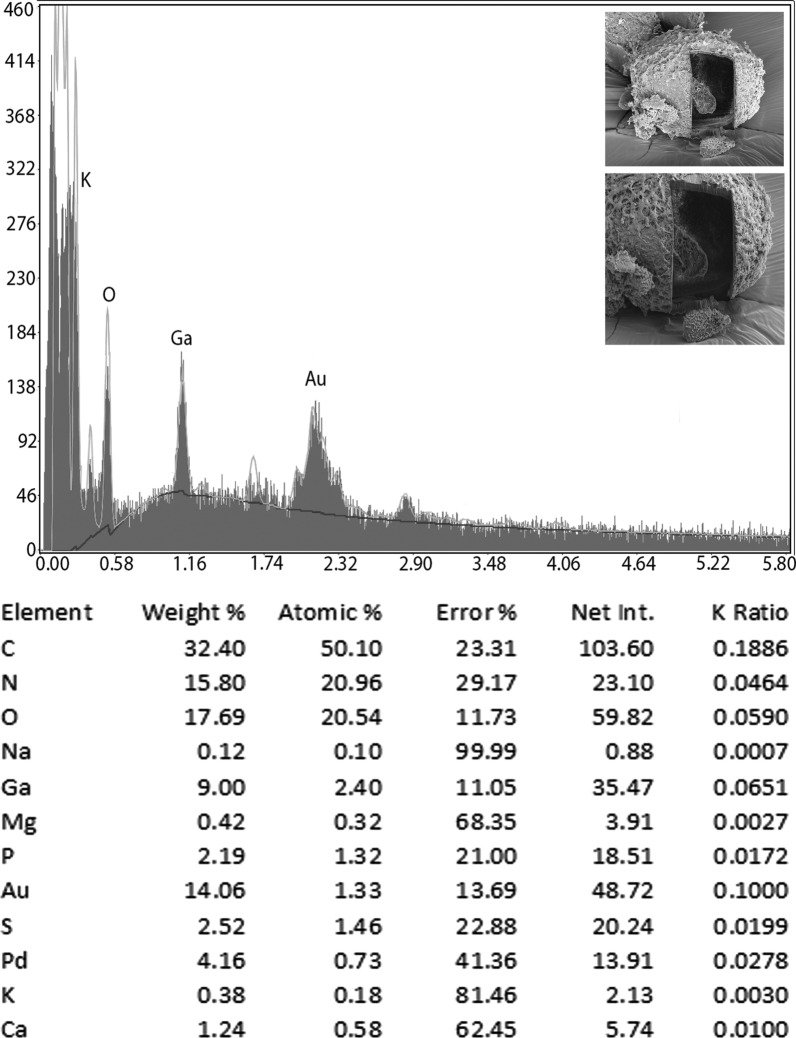
Energy-dispersive x-ray spectrum of the *Baylisascaris devosi* egg. Insert: SEM of a cross gallium cut egg

Table 2 presents the x-ray scan data for the gallium cut structures (male spike, male papillae, mouth denticles and egg) of *B. devosi*. It is a summary of the x-ray spectra given in Figs. 5, 6, 7 and 8. The worm had prominent structures. The three major chemical elements for the hardening of these structures were calcium, sulfur and phosphorus. Representative of these high levels were the denticles in the mouth region (calcium 18.56; sulfur 9.20; phosphorus 6.79 wt%).

**Table 2.** Summary of x-ray scans for *Baylisascaris devosi* structures that have been cut with a gallium beam (LMIS) and then scanned

Chemical elements	*Baylisascaris devosi*			
	Male spike	Male papillae	Mouth denticles	Egg
Sodium (Na)	0.15	1.37	0.24	0.12
Magnesium (Mg)	0.20	0.49	1.08	0.42
Phosphorus (P)	2.59	1.92	6.79	2.19
Sulfur (S)	5.07	1.39	9.20	2.52
Potasium (K)	0.39	0.00	0.43	0.38
Calcium (Ca)	6.01	2.50	18.56	1.24

Wt%. Common protoplasmic elements omitted (C, N, O) as well as preparation chemicals (Pd, Au, Ga)

### Molecular results

The specimens of *B. devosi* successfully presented amplification of the partial *Cox1*, LSU rDNA and ITS-rDNA genes. The *Cox1* dataset (679 nt) included 14 sequences for six species of genus *Baylisascaris* and the sequence of *B. devosi* obtained in the present study. The LSU rDNA dataset (998 nt) included 14 sequences for 7 species of genus *Baylisascaris* and our sequence of *B. devosi*. Also, the ITS-rDNA gene dataset (938 nt) included 15 sequences for 7 species of genus *Baylisascaris* and the sequence of *B. devosi* obtained in this study. Intra-species variation within isolates of *B. devosi* was 0.8–1.4%, 0% and 0–0.2% for *Cox1*, LSU rDNA and ITS-rDNA fragments, respectively. Inter-generic differences based on the partial *Cox1* sequence between our sequence of *B. devosi* with *B. procyonis, B. columnaris, B. transfuga, B. ailuri* and *B. schroederi* were 4.2–4.7%, 4.5–5.3%, 8.1%, 9.4% and 10.3%, respectively. Inter-generic differences based on the partial LSU rDNA sequence between our sequence of *B. devosi* with *B. procyonis, B. columnaris, B. transfuga, B. schroederi, B. ailuri* and *B. tasmaniensis* were 0.9%, 1.0%, 1.8–2.1%, 1.8%, 2.1% and 3.3%, respectively. Also, the sequence divergence based on the partial sequence of ITS-rDNA between *B. devosi* with *B. procyonis*, *B. columnaris, B. venezuelensis, B. transfuga*, *B. schroederi* and *B. tasmaniensis* was 1.2%, 1.1–1.2%, 5.3%, 2.4–2.5%, 3.6% and 3.7%, respectively.

**Fig. 9:**
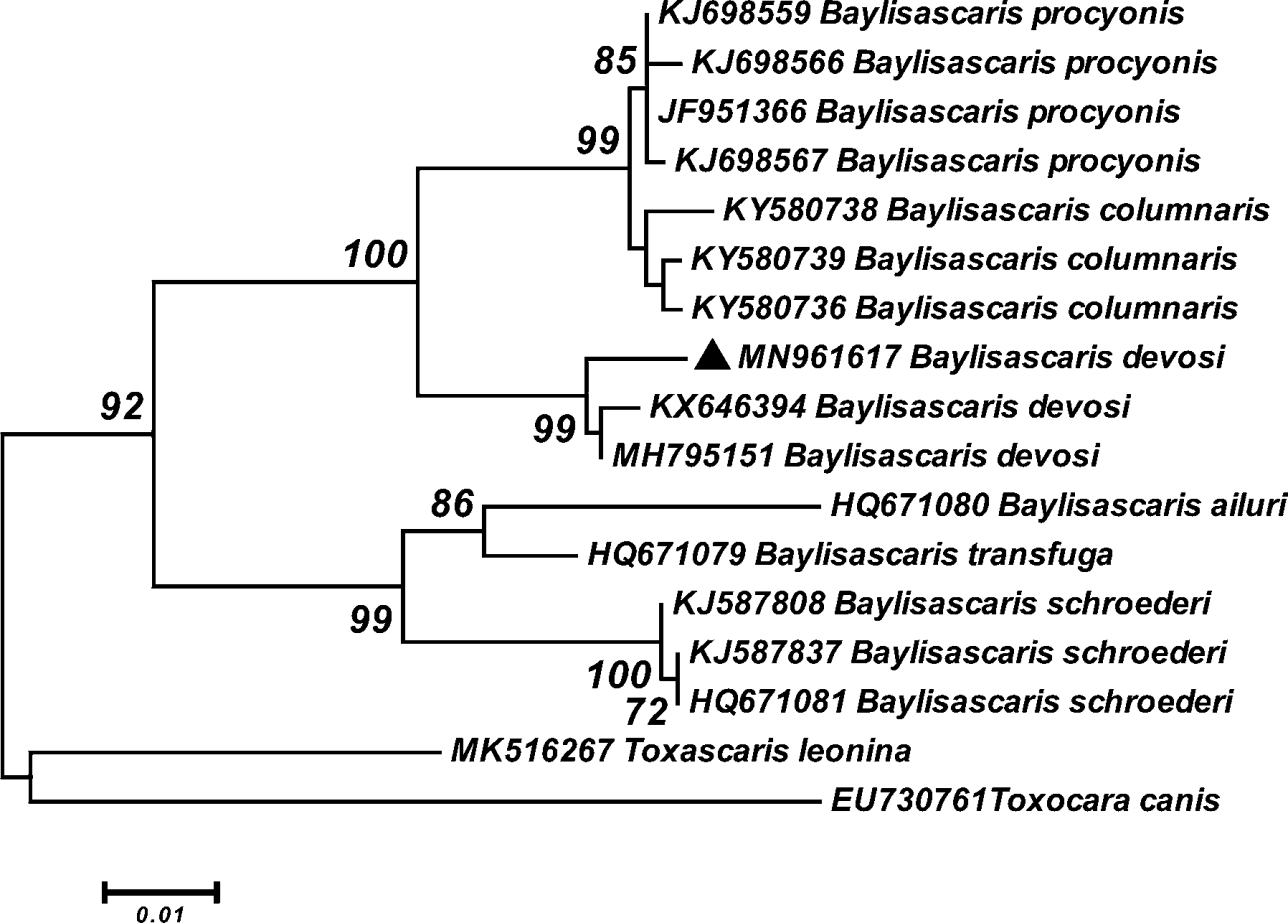
Phylogenetic analysis of the *Cox1* sequence of *B. devosi* isolate obtained in this study (black upward-pointing triangle) and reference sequences retrieved from GenBank. The tree was constructed using the maximum likelihood method and the Tamura three-parameter model in MEGA6 software. Bootstrap values < 70 are omitted. *Toxocara canis* and *Toxascaris leonina* sequences were used as outgroup

According to phylogenetic analysis based on the *Cox1* gene, our sequence of *B. devosi* (MN961617) was grouped with *B. devosi* (KX646394) isolated from sable in Russia and *B. devosi* (MH795151) isolated from fisher in Canada with strong support. This clade appeared as a sister group with *B. procyonis* (KJ698559, KJ698566, JF951366 and KJ698567) and *B. columnaris* (KY580736, KY580738 and KY580739) with statistical support of 100%. Moreover, *B. ailuri* (HQ671080),* B. transfuga* (HQ671079) and *B. schroederi* (KJ587808, KJ587837 and HQ671081) were located as the sister group of the major clade with high statistical support (Fig. 9).

**Fig. 10:**
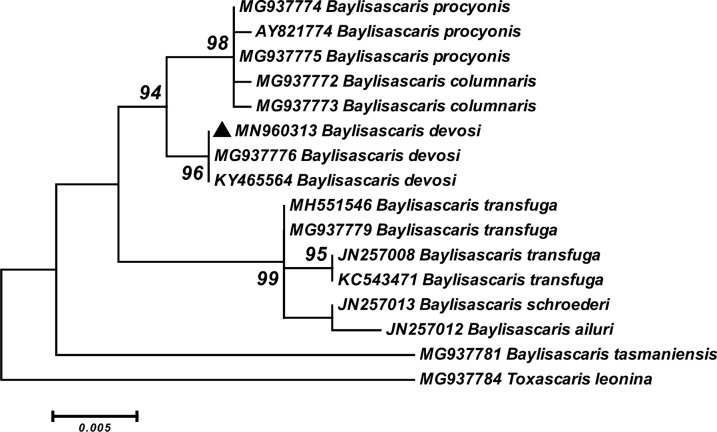
Phylogenetic analysis of LSU rDNA sequence of *B. devosi* isolate obtained in this study (black upward-pointing triangle) and reference sequences retrieved from GenBank. The tree was constructed using the maximum likelihood method and Tamura three-parameter model in MEGA6 software. Bootstrap values < 70 are omitted. The *Toxascaris leonina* sequence was used as outgroup

The phylogenetic analysis based on the LSU rDNA illustrated that the *B. devosi* sequence obtained in this study was grouped with *B. devosi* (MG937776) isolated from fisher in Canada and *B. devosi* (KY465564) isolated from sable in Russia with high bootstrap values. This clade was located as monophyletic and a sister taxon of *B. procyonis* (AY821774, MG937774 and MG937775) and *B. columnaris* (MG937772 and MG937773) with strong support. Indeed, as a major sister group, *B. transfuga* (MH551546, MG937779, JN257008 and KC543471)*, B. schroederi* (JN257013) and *B. ailuri* (JN257012) were clustered close to the clade of *B. devosi*, *B. procyonis* and *B. columnaris* in the tree. The sequence of *B. tasmaniensis* (MG937781) was located at the basal position to the members of genus *Baylisascaris* (Fig. 10).

**Fig. 11:**
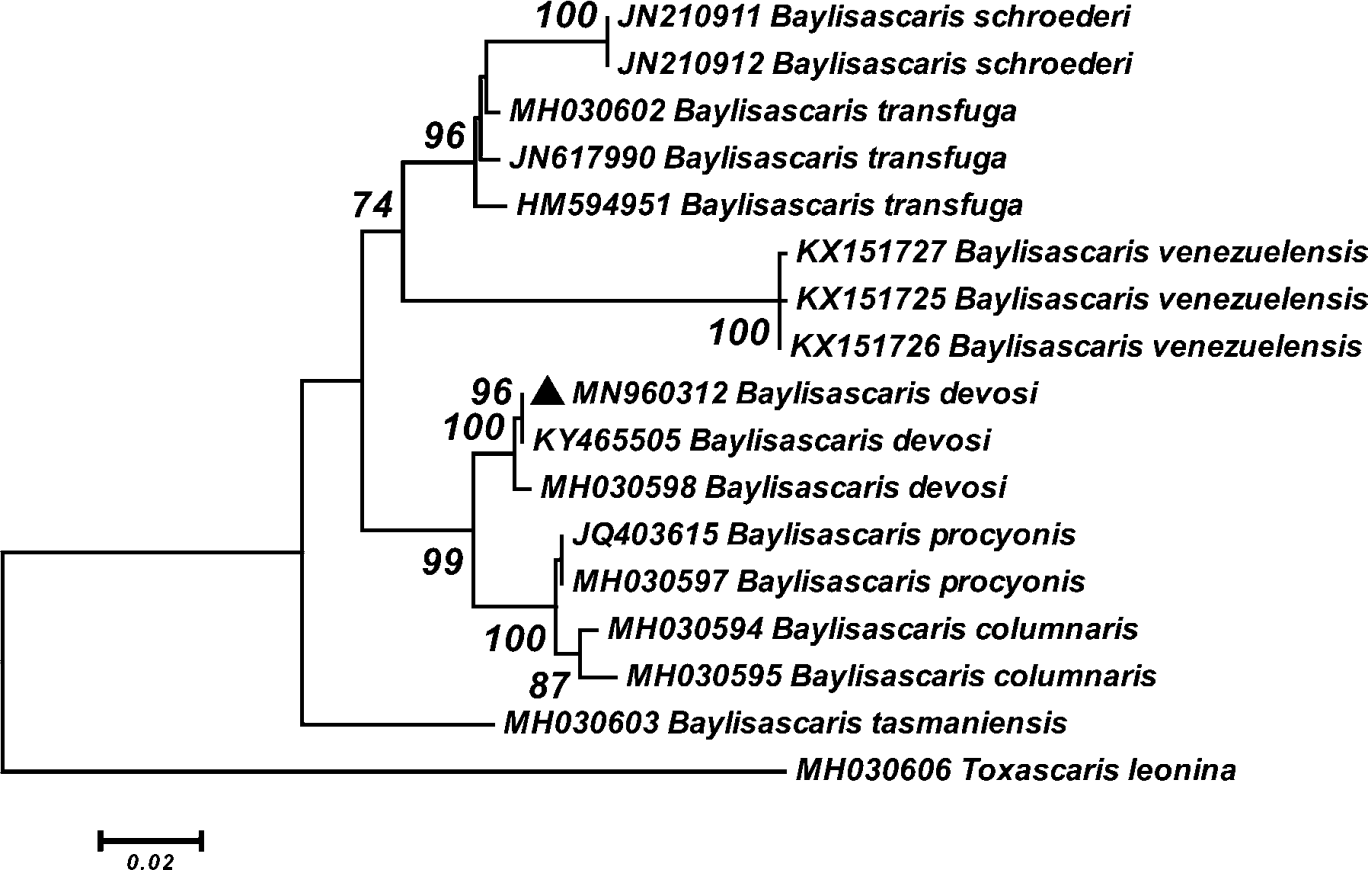
Phylogenetic analysis of the ITS sequence of *B. devosi* isolate obtained in this study (black upward-pointing triangle) and reference sequences retrieved from GenBank. The tree was constructed using the maximum likelihood method and Tamura three-parameter model in MEGA6 software. Bootstrap values < 70 are omitted. The *Toxascaris leonina* sequence was used as outgroup

The phylogenetic reconstruction based on the partial sequence of ITS-rDNA showed that our sequence of *B. devosi* was clustered with *B. devosi* (KY465505) isolated from sable in Russia and *B. devosi* (MH030598) isolated from fisher in Canada with high statistical support. This clade appeared to be a sister group consisting of *B. procyonis* (JQ403615 and MH030597) and *B. columnaris* (MH030594 and MH030595). These two clades grouped as a sister taxon with *B. schroederi* (JN210911 and JN210912), *B. transfuga* (MH030602, JN617990 and HM594951) and *B. venezuelensis* (KX151725, KX151726 and KX151727). Similar to the LSU rDNA tree, *B. tasmaniensis* (MG937781) isolate was placed at the basal position to the members of genus *Baylisascaris* (Fig. 11).

## Discussion

*Baylisascaris* species are identified according to their morphological features such as the length and width of the adult worm, length of spicules, number of pre-anal papillae and position of the cervical alae [1]. Adult males of *B. devosi* measure 57 to 123 mm in length and 1.3 mm in width, while adult females are 105–285 mm long and 2.5 mm wide. The male worms have relatively short and thick spicules (0.39–0.54 mm), the number of pre-anal papillae ranges from 21 to 40 pairs, and the cervical alae are invisible [2, 5, 9, 15]. In this study, the body length of female (80–160 mm) and male (65–120 mm) worms was similar to those in the study conducted by Tranbenkova and Spiridonov (2017), who reported the female and male body lengths were 78–168 mm and 58–140 mm, respectively [9]. In our study, a triangular mouth of *B. devosi* surrounded by three lips and armed with small denticles was observed using the scanning electron microscope. Our findings are in agreement with the reports of Tranbenkova and Spiridonov (2017); the results of morphological examination showed that adult worms had three prominent anterior lips with a row of denticles on the lip surface [9]. In the female *B. devosi* isolated from Pine marten in Iran, the vulvar opening was situated in the anterior half of the body length, and similar to our study, the vulvar opening of the female worm of Russian *B. devosi* isolated from sable was situated on the border between the first and second quarter of the body length [9]. In this study, the total count of pre-cloacal papillae was around 28 pairs.

One of the major advances in parasite research has been the use of dual-beam scanning electron microscopes and x-ray software (EDXA, energy-dispersive x-ray analysis) [16–19]. The chemical elements of a parasite structure can be determined in minute amounts and then applied to the morphology of the organism. The dual beam allows the researcher to cut the minute structures (such as hooks of acanthocephala) with a gallium beam and analyze the surface [16, 20–23]. Most of the analysis is of a qualitative, not quantitative, nature for the parasites. Often the analysis will relate to parasite nutrition and habitat. The technique may also be useful for taxonomy. Prominent structures of *B. devosi* including eggs, male spikes, mouth denticles and male papillae were cut and analyzed for chemical elements. These structures, with the exception of the eggs and male papillae, had a high level of hardening elements (Ca, P, S), which help explain the chemical nature and morphology of the worm. The cited elements are common in other hardened structures of animals such as the hooks of acanthocephala and mammalian teeth. The listed elements probably form a calcium phosphate apatite.

DNA sequence-based methods have been widely applied over recent years for species identification, classification and evaluation of phylogenetic relationships among ascarid nematodes [24, 25]. Morphological differences between *Baylisascaris* species are not so clear; therefore, utilization of the sequence data will be helpful in this aspect. Based on the results of the LSU rDNA gene analyses, there is 100% similarity between *B. devosi* obtained in this study and the isolates collected from fisher in Canada and sable in Russia. Also based on ITS-rDNA, our sequence has 100% and 99.8% homology with Russian *B. devosi* from sable and Canadian *B. devosi* from fisher, respectively. Meanwhile, based on the *Cox1* gene, our sequence illustrated more identity with Russian *B. devosi* (99.2%) than Canadian *B. devosi* (98.6%), which is in agreement with the ITS-rDNA tree. Therefore, considering these genetic markers, our sequence had the greatest similarity with Russian *B. devosi* isolated from sable. Inter-generic differences are noted between *B. devosi* and other members of species of genus *Baylisascaris* based on partial LSU rDNA, ITS-rDNA and *Cox1* genes, being 0.9%–3.3%, 1.1–5.3% and 4.2–10.3%, respectively. Due to the high level of divergence in the *Cox1* gene, it is appropriate to consider it for phylogenetic and taxonomic studies of genus *Baylisascaris*. The results of some studies confirmed that mitochondrial genes are suitable markers for phylogenetic relationships and evolutionary biological aspects within the genus *Baylisascaris* [24, 26, 27].

The reconstructed phylogenetic analysis based on LSU rDNA, ITS-rDNA and *Cox1* genes illustrated two main well-supported *Baylisascaris* clades. Within clade 1, the geographic isolates of *B. devosi* along with *B. columnaris* and *B. procyonis* formed a monophyletic taxon. The isolates of *B. procyonis* and *B. columnaris* in all trees were sisters to *B. devosi* with very high or absolute support. Clade 2, which contained the geographic isolates *B. ailuri*, *B. schroederi*, *B. transfuga* and *B. venezuelensis*, was monophyletic and sister group to Clade 1.

Many knowledge gaps still exist in the ecology of *B. devosi.* This nematode lives in the small intestine of carnivorous mammals such as different species of marten and the third-stage larvae present in the cervical and thoracic musculature of paratenic hosts such as rodents and birds [1, 2, 5]. Human is the accidental host of *B. devosi*, and baylisascariasis in human can be caused by this nematode, but the clinical diagnosis of the disease is based on the serological tests, and these tests cannot discriminate among *Baylisascaris* species [24, 28]. Until now, none of the *Baylisascaris* species have been reported from Iran, and to our knowledge this study also is the first report of *B. devosi* isolated from the Pine marten. Pine marten is the rarest of mammalian carnivore species in Iran. Baradarani et al. reported five new specimens of Pine marten from the Mazandaran and Golestan provinces within the Caspian region of Iran [29].

## Conclusions

In this study, to our knowledge for the first time, the occurrence of *B. devosi* infection is reported in Pine marten. Metal analysis distinguished high levels of phosphorus, calcium and sulfur in male spike and mouth denticle structures. Molecular analysis of partial LSU rDNA, ITS-rDNA and *Cox1* genes showed that these three regions are suitable molecular markers for inferring phylogenetic relationships of *Baylisascaris* species. Furthermore, the high divergence of *Cox1* among between *Baylisascaris* species indicates that *Cox1* could be used for the identification of species and molecular phylogenetic studies.

## Data Availability

All data generated or analyzed during the present study are included in this published article.

## Abbreviations

*Cox1*: Cyclooxygenase 1
LSU rDNA: Large subunit ribosomal DNA
ITS*-*rDNA: Internal transcribed spacer ribosomal DNA
PCR: Polymerase chain reaction
EDAX: Energy dispersive x-ray analysis
SEM: Scanning electron microscopy
